# Patterns and factors associated with dental service utilization among insured people: a data mining approach

**DOI:** 10.1186/s12911-024-02572-6

**Published:** 2024-06-24

**Authors:** Zahra Pouraskari, Reza Yazdani, Maryam Khademi, Hossein Hessari

**Affiliations:** 1 Department of Community Oral Health, School of Dentistry, Tehran University of Medical Sciences, Tehran, Iran; 2 Research Centre for Caries Prevention, Dentistry Research Institute, Tehran University of Medical Sciences, Tehran, Iran; 3 Department of Applied Mathematics, South Tehran Branch, Islamic Azad University, Tehran, Iran

**Keywords:** Dental insurance, Dental service, Secondary data analysis, Data mining, Utilization, CRISP

## Abstract

**Background:**

Insurance databases contain valuable information related to the use of dental services. This data is instrumental in decision-making processes, enhancing risk assessment, and predicting outcomes. The objective of this study was to identify patterns and factors influencing the utilization of dental services among complementary insured individuals, employing a data mining methodology.

**Methods:**

A secondary data analysis was conducted using a dental insurance dataset from Iran in 2022. The Cross-Industry Standard Process for Data Mining (CRISP-DM) was employed as a data mining approach for knowledge extraction from the database. The utilization of dental services was the outcome of interest, and independent variables were chosen based on the available information in the insurance dataset. Dental services were categorized into nine groups: diagnostic, preventive, periodontal, restorative, endodontic, prosthetic, implant, extraction/surgical, and orthodontic procedures. The independent variables included age, gender, family size, insurance history, franchise, insurance limit, and policyholder. A multinomial logistic regression model was utilized to investigate the factors associated with dental care utilization. All analyses were conducted using RapidMiner Version 2020.

**Results:**

The analysis encompassed a total of 654,418 records, corresponding to 118,268 insured individuals. Predominantly, restorative treatments were the most utilized services, accounting for approximately 38% of all services, followed by diagnostic (18.35%) and endodontic (13.3%) care. Individuals aged between 36 and 60 years had the highest rate of utilization for any dental services. Additionally, families comprising three to four members, individuals with a one-year insurance history, people contracted with a 20% franchise, individuals with a high insurance limit, and insured individuals with a small policyholder, exhibited the highest rate of service usage compared to their counterparts. The regression model revealed that all independent variables were significantly associated with the use of dental services. However, the patterns of association varied among different service categories.

**Conclusions:**

Restorative treatments emerged as the most frequently used dental services among insured individuals, followed by diagnostic and endodontic procedures. The pattern of service utilization was influenced by the characteristics of the insured individuals and attributes related to their insurance.

## Background

The World Health Organization has outlined a framework for health systems that comprises six building blocks: service delivery, health workforce, health information systems, access to essential medicines, financing, and leadership/governance. This framework includes intermediate objectives, such as access, coverage, quality, and safety. The overarching goals of this framework are to improve health (both in terms of level and equity), responsiveness, social and financial risk protection, and efficiency [1].

Both public and private health insurance are pivotal in financing health systems and providing coverage for health services. They serve as significant mechanisms for financial protection, mitigating uncertainty and financial risk tied to healthcare costs [2]. Furthermore, health insurance can enhance prompt access to services, ultimately leading to health promotion in the context of service delivery. Service delivery, as an immediate output of health systems, reflects the availability and distribution of care [1]. Consequently, a substantial portion of the information pertaining to the provision of services and their associated costs is allocated to health insurance.

Insurance databases serve as important secondary data sources, typically gathered from payments made for healthcare services [3]. These databases encompass details about the services provided, including the International Classification of Diseases (ICDs), types of dental services, expenditures, dates, length of enrollment, and basic sociodemographic characteristics of the insured. These characteristics include age, gender, area of residence, race/ethnicity, and income [4–6].

Insurance databases aid insurance companies in decision-making processes by predicting demand, formulating future strategies, and discerning customer preferences [7]. Furthermore, healthcare systems can leverage this data to enhance the effectiveness and quality of services, advance risk assessment and disease prevention strategies, and predict outcomes [8].

Insurance data has been utilized in prior studies to investigate patterns of early childhood dental care utilization [9], evaluate the frequency and types of dental care used in various communities [4, 5, 10, 11], examine regional and gender disparities in oral health [10], and assess the quality of dental care [12]. Several studies have reported that a variety of sociodemographic, regional, and general health factors are associated with the utilization of dental care among insured individuals [4, 5, 10]. Furthermore, the type of services utilized by the insured individuals varies according to different dental plans and benefit packages [11]. Moreover, findings from other studies have indicated that regular preventive visits can reduce costs among insured children [9]. Also, there has been a slight improvement in the overall quality of dental services over the years, particularly among insured children aged 0–5 years [12].

Previous investigations in Iran suggested that people with both public and private health insurance more frequently used restorative and expensive dental services compared to those who only had public health insurance [13]. Furthermore, adolescents with complementary health insurance exhibited a higher prevalence of decayed and filled teeth, despite their increased utilization of dental services compared to their counterparts [14]. The Central Insurance Organization (CIO) of Iran annually publishes general statistics of complementary health insurance, including information on growth rate, premium production, the number of insured individuals, and the loss ratio. However, this annual report does not provide specific details or information related to dental services and their associated costs.

There are two types of public and complementary (private) dental insurance in Iran. All of the public insurances cover only the basic package of oral healthcare. The majority of dental services are provided through complementary insurance [15]. Generally, there are 26 private insurance companies that offer complementary dental insurance. Each company maintains a separate claims database that includes service-related information, such as the total cost of the service, the payment made by the insurer, the franchise, the insurance limit, and the date of service delivery. Additionally, these databases contain sociodemographic characteristics of the insured individuals, including age, gender, and family size.

To the best of our knowledge, no study has yet examined the utilization of dental services among beneficiaries of complementary insurance using insurance claims data in Iran. The current study aimed to assess the patterns and associated factors of dental service utilization among individuals with complementary insurance, employing a data mining methodology, using dental claims data from 2018 to 2021.

## Methods

### Study design

This research, a secondary data analysis, was conducted as a cross-sectional study. The Ethics Committee of the School of Dentistry at Tehran University of Medical Sciences granted approval for this study (IR.TUMS.DENTISTRY.REC.1399.241).

The claims data was sourced from a big private company that serves as an intermediary for private insurers concerning dental plans in complementary health insurance. At the time the study was conducted, the dataset contained information related to dental services provided to insured individuals under an insurance contract with seven private companies. A formal letter indicating the objectives and benefits of study was sent to the company for participation in the study. The company accepted and showed interest for further cooperation through a written informed consent.

This database included approximately 2000 dental contracts from a variety of policyholders, and contained details related to the service, such as the total cost of the service, the reimbursement from the insurer, franchise (co-insurance), the insurance limit, and the date of service delivery. Additionally, the database included certain sociodemographic characteristics of the insured individuals, such as age, gender, and family size. All the available data were included in the study.

The Cross-Industry Standard Process for Data Mining (CRISP-DM) was employed as a data mining methodology for extracting knowledge from the insurance database. The primary objective was to predict the utilization of dental services and the associated factors using a regression model based on existing historical patterns. A comprehensive sample of dental services provided to insured individuals was examined. No additional eligibility criteria were established, and no formal sample size estimation was conducted.

### Statistical analysis

**Fig. 1:**
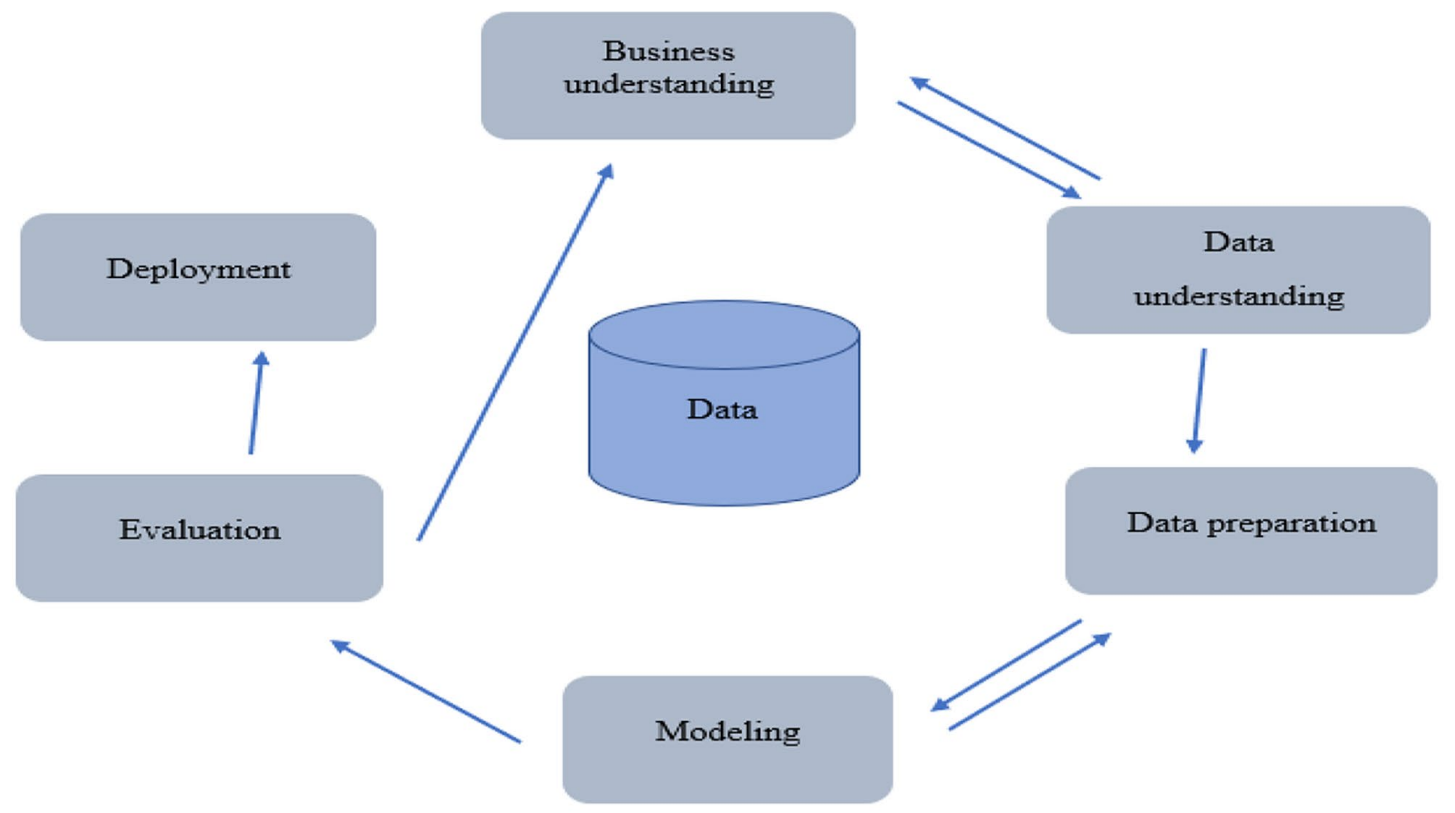
Steps of the CRISP-DM The figure is produced by the authors

The CRISP-DM composed of six steps as follows: (1) business understanding, (2) data understanding, (3) data preparation, (4) modeling, (5) evaluation, and (6) implementation. Figure 1 shows the different steps of CRISP-DM.

#### Business understanding

Predicting the utilization of dental services is vital for assessing the treatment needs of insured individuals and for planning future service provision by insurance companies. For this analysis, we utilized insurance claims data, which was provided in the form of a Microsoft Excel file. Data analysis was conducted using RapidMiner.

#### Data understanding

The claims data of insured individuals between April 16, 2018, and May 16, 2021, was utilized for this study. This database was procured from a private company that serves as a consultant for dental contracts of private insurers. The dataset comprised 655,564 records of dental services provided to the insured. Only records that contained complete information about the service provided were used. Data attributes for the utilization of dental services were selected from the claims database. A total of 16 attributes were included and analyzed during the data preparation phase. These attributes are listed in Table 1. The first five attributes pertain to the characteristics of the insured individuals, while the remaining attributes are related to service delivery and the insurance contract.

**Table 1:** List of attributes related to the utilization of dental services (attributes used in the regression model are bolded)

	Attribute	Description
	Insured characteristics	
1	Age	Age of the insured person at the time of receiving the service
2	Gender	Insured gender
3	Family size	Number of persons in each household
4	Insurance history	History of complementary health insurance in years
5	Patient ID	Identification ID used for registration of insured people
	Service-related attributes	
6	Tooth number	Number of treated teeth based on the universal numbering system
7	Service code	The code assigned for each service based on the national classification of dental services (considered as the target attribute)
8	Date of service delivery	Date on which the service was provided for the insured person
	Insurance-related attributes	
9	Service tariff	Price of dental service set by the insurance company
10	Family insurance limit	Total insurance limit for all household members
11	Individual insurance limit	Total insurance limit for each insured person
12	Insurer payment	Cost share of insurance company for dental services
13	Insurer code	Special code defined for each insurer by the mediator company
14	Franchise	Cost share of insured people for dental services
15	Policyholder	Person or institution who has an insurance policy with an insurance company
16	Start date of insurance contract	Start date of dental service provision for the insured

#### Data preparation

An explanatory data analysis was conducted using the RapidMiner Software. Additionally, preprocessing of the original dataset was carried out, which included cleaning, removing redundant attributes, imputing missing values, and transforming attributes. After the cleaning process, the dataset contained 654,418 records, corresponding to 118,268 insured individuals. A total of 1,146 incomplete records were excluded from the dataset. Certain initial attributes, such as patient ID, the insurer code, date of service delivery, and the start date of the insurance contract, were removed as they were deemed unnecessary for the data analysis. Additionally, the insurer payment and service tariff were excluded from the analysis, as the study did not focus on the cost of services. There were only a few instances of having a family insurance limit, as well as having more than one insurer; therefore, these two attributes were also excluded from the analysis.

We transformed and/or relabeled some other variables. The age of the insured individuals was categorized into five groups: <12, 13–19, 20–35, 36–60, and ≥ 61 years. Family size was analyzed as a categorical variable based on the number of persons in each household, categorized as follows: 1–2 persons, 3–4 persons, and ≥ 5 persons. Moreover, we considered three categories for insurance history: 1, 2, and ≥ 3 years.

In terms of the franchise (a fixed percentage that an insured person must pay toward a covered service), there were three groups: no franchise, 10% franchise, and 20% franchise. The individual insurance limit was divided into three levels: up to 10 million IRRs (low), between 10 and 30 million IRRs (medium), and more than 30 million IRRs (high). Additionally, policyholders were categorized into three groups based on the number of insured people: small (< 1000 insured), medium (1001–5000 insured), and large (> 5000 insured). The service codes were transformed into a service category, which was considered as the outcome variable. These categories were based on the national classification of dental services, including: (1) diagnosis (oral examination and radiography), (2) preventive, (3) periodontal, (4) restorative, (5) endodontic, (6) prosthetic, (7) implant, (8) extraction and surgical, and (9) orthodontic.

#### Modeling

The pattern of dental service utilization was extracted based on the insured characteristics, service-related variables, and attributes pertained to insurance contract. We employed multinomial logistic regression to assess the utilization of dental services and to investigate the relationships between independent and dependent variables. For each independent variable incorporated into the model, the category with the highest frequency was chosen as the reference group. In terms of the dependent variable, preventive services were used as the reference category and were compared with other categories.

#### Evaluation

The evaluation phase is a crucial component of the data analysis process. For predicting the utilization of dental services, the model’s performance was evaluated based on the model fitting criteria. Table 2 illustrate the model fitting information for multinomial logistic regression in our data mining process.

**Table 2:** The model fitting information for multinomial logistic regression

Model	Model Fitting Criteria			Likelihood Ratio Tests		
	AIC	BIC	-2 Log Likelihood	Chi-Square	df	Sig
Intercept only	179938.53	180029.66	179922.53			
Final	81997.52	83455.64	81741.52	98,181	120	0.000

AIC: Akaike’s Information Criterion, BIC: Bayesian Information Criterion, df: degree of freedom

#### Deployment

The deployment phase entailed applying the insights and predictions obtained from the modeling and evaluation stages. In the context of the utilization of dental services, findings of the present study could be beneficial to predict usage in future. Proper coverage of dental services by the insurance companies encourages people to purchase complementary health insurance. Moreover, providing dental coverage according to the specific insured characteristics and the pattern of service utilization could be employed in designing suitable benefit package. Consequently, this analysis is helpful for insurers to promote client satisfaction and informed decision making.

## Results

A comprehensive analysis was conducted on 654,418 records, which corresponded to 118,268 insured individuals. Approximately 54% of the services were associated with female insured people. The majority of services (about 45.4%) were utilized by insured individuals aged 36 to 60 years. Additionally, more than half of the services were provided to families with three or four members. Regarding insurance history, approximately 38% of the services were attributed to individuals who had a one-year history of complementary dental insurance.

Considering the concept of franchise, the utilization rate was the lowest for contracts with no franchise, while the frequencies for 10% and 20% franchise were nearly equal. Furthermore, insurance contracts with a medium cost cap accounted for approximately 55% of services. When compared to medium and large policyholders, small policyholders utilized the most services. Overall, restorations were the most common services, making up 37.6% of total services, followed by diagnostic services at 18.3% and endodontic treatments at 13.3%. Orthodontic treatments had the lowest frequency, with a utilization rate of less than 1% (0.5%). The utilization rates of dental services based on independent attributes are detailed in Table 3.

**Table 3:** Utilization of dental services according to the insured people characteristics, service-related and insurance-related variables

		Type of service Number (Percent)									
		Any services	Diagnosis	Preventive	Periodontal	Restoration	Endodontic	Prosthetic	Implant	Surgical/Extraction	Orthodontic
Total		654,418	120,070 (18.35)	7039 (1.08)	38,385 (5.87)	246,103 (37.60)	86,994 (13.30)	69,157 (10.57)	21,455 (3.30)	61,837 (9.45)	3378 (0.5)
Age (Year)	0–12	64,194 (9.8)	(10.1)	(9.6)	(0.6)	(42.6)	(20.9)	(0.2)	(0.0)	(14.3)	(1.6)
	13–19	24,743 (3.8)	(16.5)	(2.6)	(6.2)	(50.9)	(9.3)	(1.7)	(0.2)	(8.0)	(4.6)
	20–35	219,649 (33.6)	(21.5)	(0.0)	(6.2)	(40.5)	(13.6)	(8.5)	(1.2)	(8.0)	(0.4)
	36–60	296,987 (45.4)	(18.6)	(0.0)	(6.7)	(35.1)	(12.4)	(13.6)	(4.7)	(8.8)	(0.1)
	61–100	48,845 (7.5)	(14.6)	(0.0)	(5.8)	(26.9)	(9.2)	(19.3)	(10.0)	(14.2)	(0.0)
Gender	man	315,422 (48.2)	(18.1)	(1.1)	(5.8)	(36.3)	(13.5)	(10.9)	(3.4)	(10.4)	(0.4)
	woman	338,996 (51.8)	(18.6)	(1.0)	(5.9)	(38.8)	(13.1)	(10.2)	(3.1)	(8.6)	(0.6)
Family size	1–2	217,812 (33.3)	(18.8)	(0.2)	(6.5)	(37.5)	(12.7)	(11.6)	(3.6)	(8.8)	(0.3)
	3–4	354,807 (54.2)	(18.4)	(1.5)	(5.5)	(37.7)	(13.6)	(10.1)	(3.1)	(9.5)	(0.6)
	5–11	81,799 (12.5)	(16.9)	(1.7)	(5.6)	(37.6)	(13.7)	(9.8)	(3.1)	(10.9)	(0.8)
Insurance history (Year)	1	249,155 (38.1)	(17.8)	(0.9)	(5.7)	(38.1)	(13.4)	(10.7)	(3.3)	(9.7)	(0.5)
	2	197,812 (30.2)	(17.5)	(1.2)	(5.7)	(38.1)	(13.9)	(10.2)	(2.7)	(10.2)	(0.5)
	≥ 3	207,451 (31.7)	(19.9)	(1.1)	(6.2)	(36.6)	(12.6)	(10.7)	(3.7)	(8.5)	(0.6)
Franchise	0	64,538 (9.9)	(23.8)	(0.5)	(4.4)	(34.2)	(11.5)	(12.7)	(3.4)	(9.0)	(0.3)
	10%	293,819 (44.9)	(16.0)	(1.3)	(6.9)	(38.0)	(13.7)	(10.5)	(3.3)	(9.8)	(0.6)
	20%	296,061 (45.2)	(19.5)	(1.0)	(5.2)	(38.0)	(13.3)	(10.1)	(3.2)	(9.2)	(0.5)
Insurance limit	Low	94,452 (14.4)	(18.2)	(1.1)	(6.1)	(37.4)	(12.9)	(10.9)	(3.6)	(9.3)	(0.5)
	medium	358,256 (54.7)	(17.8)	(1.2)	(6.0)	(37.7)	(13.0)	(10.7)	(3.6)	(9.3)	(0.5)
	High	201,710 (30.8)	(19.3)	(0.9)	(5.4)	(37.5)	(14.0)	(10.0)	(2.6)	(10.0)	(0.5)
Policyholder	Small	242,765 (37.1)	(16.9)	(1.2)	(6.2)	(38.9)	(13.9)	(10.2)	(2.9)	(9.4)	(0.5)
	Medium	209,765 (32.1)	(19.2)	(1.1)	(5.4)	(37.6)	(13.6)	(10.3)	(2.6)	(9.7)	(0.5)
	Large	201,888 (30.9)	(19.2)	(0.9)	(6.0)	(36.1)	(12.2)	(11.3)	(4.4)	(9.2)	(0.6)

The multinomial logistic regression analysis revealed that individuals under the age of 12 years had the lowest probability of utilizing all service categories (*P* < 0.001). The highest odds ratio for implant usage was observed among insured individuals who were 61 years or older (OR: 1.8, 95% CI: 1.13–2.88). Furthermore, individuals aged 20–35 years had the highest likelihood of undergoing orthodontic procedures (OR: 6.55, 95% CI: 4.74–9.06). The regression model also indicated that men significantly received more prosthetic (*P* < 0.01) and extraction/surgical procedures (*P* < 0.001), while they were less likely to opt for orthodontic treatments (*P* < 0.001).

The probability of utilizing periodontal treatments, restorations, root canal therapies, orthodontic treatments, and extraction/surgical services was significantly higher among households with five or more members. As the duration of dental coverage increased, the likelihood of using restorations, endodontic therapies, and extraction/surgical procedures significantly decreased. However, individuals with the longest history of insurance significantly utilized more orthodontic services. Insured individuals who had an insurance policy without a franchise were more likely to receive diagnostic and prosthetic care (*P* < 0.001). Having a high insurance limit was associated with a higher usage of all dental services, except for implants. In terms of policyholders, those with the largest insured population were more likely to utilize periodontal therapies, orthodontic services, and implants compared to others. However, medium policyholders had the highest utilization rate of diagnostic and surgical services. More details of the multinomial logistic regression are demonstrated in Table 4.

**Table 4:** Multinomial logistic regression of factors associated with utilization of dental services among complementary insured people

		Service category															
		Diagnosis		Periodontal		Restorative		Endodontic		Prosthetic		Implant		Surgical/Extraction		Orthodontic	
		OR	95% CI	OR	95% CI	OR	95% CI	OR	95% CI	OR	95% CI	OR	95% CI	OR	95% CI	OR	95% CI
Age	0–12	0.002^***^	0.002–0.003	0.000^***^	0.000–0.000	0.005^***^	0.004–0.006	0.007^***^	0.006–0.008	0.000^***^	0.000–0.000	0.000^***^	0.000–0.000	0.007^***^	0.005–0.008	0.09^***^	0.071–0.114
	13–19	0.013^***^	0.011–0.016	0.014^***^	0.011–0.017	0.021^***^	0.018–0.026	0.011^***^	0.009–0.013	0.002^***^	0.001–0.002	0.001^***^	0.000- 0.001	0.013^***^	0.011–0.016	0.897	0.700–1.150
	20–35	1.297	0.972–1.732	1.049	0.785–1.401	1.297	0.971–1.731	1.238	0.927–1.652	0.709^*^	0.531–0.942	0.284^***^	0.212–0.380	1.051	0.787–1.404	6.551^***^	4.738–9.056
	36–60	Ref															
	≥ 61	0.652	0.409–1.039	0.749	0.470–1.195	0.673	0.423–1.073	0.659	0.413–1.050	1.221	0.766–1.945	1.809^*^	1.134–2.884	1.433	0.899–2.284	0.188^***^	0.081–0.437
Gender	Man	1.015	0.965–1.067	1.012	0.959–1.067	0.963	0.917–1.012	1.045	0.993–1.099	1.064^*^	1.011–1.121	1.052	0.994–1.113	1.215^***^	1.154–1.279	0.673^***^	0.618–0.733
	Woman	Ref															
Family size	1–2	0.978	0.872–1.097	1.071	0.954–1.202	1.037	0.925–1.162	1	0.982–1.121	1.012	0.902–1.135	1.021	0.908–1.149	0.986	0.879–1.107	0.970	0.833–1.130
	3–4	Ref															
	≥ 5	1.040	0.975–1.109	1.094^*^	1.020–1.174	1.076^*^	1.011–1.146	1.092^**^	1.024–1.165	1.056	0.987–1.129	0.997	0.924–1.076	1.194^***^	1.119–1.275	1.154^**^	1.037–1.285
Insurance history (Year)	1	Ref															
	2	0.909^**^	0.853–0.969	0.859^***^	0.803–0.920	0.843^***^	0.791–0.897	0.859^***^	0.806–0.916	0.933^*^	0.874–0.997	0.830^***^	0.772–0.893	0.927^*^	0.987–0.989	0.913	0.819–1.017
	≥ 3	1.029	0.966–1.096	0.897^**^	0.840–0.959	0.810^***^	0.761–0.861	0.778^***^	0.730–0.828	0.940	0.881–1.003	1.026	0.957–1.101	0.742^***^	0.696–0.791	1.114^*^	1.005–1.234
Franchise	0	1.60^***^	1.408–1.819	0.985	0.862–1.126	1.130	0.995–1.283	1.120	0.985–1.274	1.302^***^	1.143–1.483	0.833^**^	0.727–0.956	1.097	0.963–1.249	0.970	0.797–1.179
	10%	0.755^***^	0.716–0.797	1.204^***^	1.137–1.274	0.904^***^	0.858–0.953	0.925^**^	0.877–0.976	0.916^**^	0.867–0.968	0.850^***^	0.800-0.903	0.951	0.901–1.005	1.095^*^	1.002–1.197
	20%	Ref															
Insurance limit	low	1.092^*^	1.015–1.175	1.077	0.997–1.164	1.065	0.992–1.146	1.059	0.984–1.139	1.072	0.995–1.156	1.042	0.961–1.131	1.064	0.987–1.146	0.991	0.876–1.120
	medium	Ref															
	high	1.390^***^	1.309–1.475	1.175^***^	1.103–1.251	1.280^***^	1.207–1.357	1.371^***^	1.292–1.456	1.246^***^	1.172–1.325	1.033	0.966–1.105	1.389^***^	1.307–1.475	1.159^**^	1.052–1.277
Policyholder	Small	Ref															
	Medium	1.24^***^	1.168–1.317	1.041	0.977–1.109	1.109^**^	1.046–1.176	1.136^***^	1.070–1.206	1.167^***^	1.097–1.241	1.058	0.988–1.134	1.193^***^	1.123–1.268	1.027	0.927–1.139
	Large	1.200^***^	1.123–1.282	1.209^***^	1.128–1.297	1.087^*^	1.019–1.160	1.054	0.986–1.126	1.168^***^	1.092–1.250	1.488^***^	1.383–1.601	1.142^***^	1.068–1.222	1.611^***^	1.145–1.790

The reference group is preventive

^***^P < 0.001, ^**^P < 0.01, ^*^P < 0.05

## Discussion

The objective of this study was to evaluate the utilization of dental services and the factors associated with it using dental insurance claims. Our findings indicated that restorative services were the most frequently provided dental care for individuals with complementary insurance, regardless of age, gender, insurance history, and other independent variables. Among all services, orthodontic therapies had the lowest rate of utilization. A prior national study suggested that individuals with both public and complementary dental insurance utilized high-cost dental treatments and restorations more than other services. Furthermore, this study revealed that preventive care was the least commonly received service [13]. Contrarily, a prior systematic review on the utilization of dental services in Australia indicated that 83.5% of insured individuals had a history of using scaling services. The service with the least frequency was dentures, with a utilization rate of 3.2% [16].

Our results indicated that insured individuals aged 36–60 years had the highest rate of utilization for all dental services. This observation aligns with the findings of a previous national study [13]. Moreover, our results suggested that restorative treatments were the most commonly used services across all age groups. This observation is corroborated by previous studies that examined insured individuals from various age groups [10, 13, 17, 18]. On the other hand, our findings differ from those of some other studies. For instance, Schwendicke et al. reported that oral examinations had the highest utilization rate among very elderly insured individuals, followed by preventive and restorative services [5]. Similarly, two studies conducted by Agrasuta et al. in 2018 and Kadhium et al. in 2022 reported a variety of commonly used dental care services across different age groups [19, 20].

Insured individuals who were under 12 years old had the lowest probability of utilizing all dental procedures. The highest likelihood of using prosthetic treatments, extraction/surgical procedures, and implants was observed among the elderly insured. This finding is confirmed by Agrasuta et al., who reported the highest utilization rate of extraction and prosthetic treatments among individuals aged 65 years or older [19]. Individuals aged between 20 and 35 years significantly utilized more orthodontic treatments compared to other age groups. However, this finding contradicts the findings reported by Kadhium et al., which reported that the highest frequency of orthodontic care was observed in patients aged between 10 and 19 years [20].

Overall, women utilized more dental services than men, which is consistent with previous studies [5, 21]. In our study, both male and female participants most commonly utilized restorative services, followed by diagnostic services and endodontic treatments. This finding contradicts the results of a study by Schwendicke et al., which showed that oral examinations, preventive services, and surgical services were the most common among women, while for men, the most common services were examinations, preventive care, and restorative treatments [5]. Conversely, a national study conducted on Iranian adults in 2017 reported that the majority of both men and women predominantly utilized high-cost dental services and restorative treatments over other dental procedures [13].

In this study, it was found that men significantly utilized more prosthetic and surgical/extraction procedures, while women were more likely to undergo orthodontic therapies. This finding is in contrast with the results of a study by Bayat et al., which revealed significant differences between genders in terms of restorative services and checkups. Meanwhile, Bayat et al. found that both genders had the same likelihood of utilizing high-cost services, extractions, and preventive services [13]. Moreover, a prior study conducted in Germany indicated that while women utilized dental services nearly twice as much as men, there was no significant correlation between gender and the usage of services [5]. However, our findings are corroborated by Manski et al., who reported gender differences in the utilization of major dental care (defined as gum treatment, tooth extraction or surgery, filling, prosthesis, implants, and root canal therapies) in the United States [17].

In our study, the majority of services were utilized by households with three or four members. Families with five or more members had the highest likelihood of receiving periodontal treatments, restorations, root canal therapies, extractions/surgeries, and orthodontic treatments. However, there was no significant association between the number of family members and the utilization of other service categories. A previous study reported that families with two members had the highest overall rate of dental service utilization among those with dental care coverage in the United States. Single-person families and households with three or more members had less than half the usage rate of two-person households. These findings are not in line with our study regarding the general utilization of dental care. However, the use of various dental care categories was not evaluated in the aforementioned study [22].

In terms of insurance history, the majority of dental services were provided to individuals with one year of dental coverage. As the duration of dental insurance increased, the utilization rate decreased for all dental services, with the exception of diagnostic services, implants, and orthodontic treatments. However, this increase was only significant for orthodontic services. A previous study reported that children who were enrolled for a full year were more likely to use preventive or treatment dental care than those enrolled for part of the year. Nevertheless, this study did not evaluate the type of treatment services used [23].

In terms of franchise, the majority of total service utilization was associated with insurance contracts with a 20% franchise. Clients who had an insurance contract without a franchise were more likely to receive diagnostic and prosthetic services. Conversely, individuals with 10% co-payments utilized more periodontal and orthodontic treatments. A recent study reported a varied distribution in the utilization of dental services following a policy change that involved lower out-of-pocket payments for older insured individuals. This study showed a decrease in oral examinations, preventive care, periodontal treatment, and extractions. However, the usage of dentures and implants increased [24]. Another study conducted in the Netherlands in 2020 revealed that an increase in co-payment for dental coverage led to a significant decrease in the utilization of oral examinations, radiographs, preventive services, and direct restorations among insured individuals aged 18 years or older. Nevertheless, the usage of scaling and extraction procedures increased. For insured individuals under the age of 18 years, the pattern was similar, except for fluoride applications and radiographs, which saw an increase of 2% and 4% respectively after the co-payment increase [25].

In terms of the insurance limit, the majority of dental services were utilized by insured individuals with a medium insurance limit. We found that those with a high insurance limit were more likely to receive all categories of dental care, excluding dental implants. This aligns with a study conducted by Teusner et al. in 2017, which showed that insured individuals with a higher level of coverage utilized filling and scaling services more than those with low or medium levels of coverage. However, the highest frequency of dental extractions was reported for insured individuals with low coverage levels, which contradicts our results [11].

According to policyholders’ classification, the highest usage of any dental services was attributed to small policyholders. Large policyholders were most likely to use implants, periodontal care, and orthodontic treatments. Meanwhile, insured individuals with medium policyholders were most likely to receive endodontic treatments, surgical/extraction procedures, and diagnostic services. Our findings are largely consistent with those of Srimuang et al., who reported that individuals with generous dental benefits coverage tend to use preventive dental treatments and necessary treatments, as well as costly restorative dental treatments, more than those with lower coverage. Furthermore, this survey showed that the type of dental services used by insured individuals was influenced by the type of insurance and the extent of service coverage. In this regard, individuals covered by a social security scheme utilized more extractions and fillings, while they had the minimum usage of orthodontic treatments, fluoride applications, and dentures. Moreover, this study showed that individuals with private insurance and those insured by their employers had the least frequency of utilization for all dental care categories [26].

### Strengths and limitations

Our study, which was the first of its kind in the field of dentistry and complementary insurance in Iran, presented the status of and factors associated with the utilization of dental services among privately insured individuals. The study utilized an insurance database comprising a large population of insured individuals. This large sample size facilitated the measurement of the utilization rate and associated factors with high statistical power. Furthermore, our data included information regarding the age and gender of the insured individuals. Consequently, this study could prove beneficial for the appropriate design of complementary dental coverage, taking into account the specific needs and patterns of care usage of different age and gender groups based on the past years’ data. Additionally, the classification of services aids in providing dental plans through complementary health insurance based on the most frequently utilized procedures.

However, our study had some limitations. Firstly, individuals with complementary insurance may have different socioeconomic and oral health statuses compared to the general population. Therefore, the results of this study should be interpreted with caution and may only apply to individuals with complementary dental insurance. Secondly, we lacked information about insured individuals who did not have a history of dental service utilization. This prevented us from making a comparison between our study population and their counterparts in terms of demographic characteristics and insurance-related variables. Thirdly, our data was based on the utilization of services and did not include any socioeconomic features of the insured population. As a result, we were unable to evaluate the association between the socioeconomic status of the insured individuals and the utilization of services. Finally, the type and extent of dental services may vary among different contracts. Therefore, insured individuals might refuse to seek services that are not covered by their insurance policy, which could affect the observed differences in the utilization of various dental procedures.

## Conclusions

Our study found that the majority of individuals covered by complementary health insurance utilized dental restorations, irrespective of their age, gender, family size, and insurance-related attributes, such as co-insurance, insurance limit, and type of policyholder. Preventive services accounted for only about 1% of the total services provided to the insured. The proportion of each type of dental care varied according to personal characteristics and insurance-related specifics. Therefore, these variations should be taken into account when designing more flexible and equitable dental plans. The inclusion of appropriate preventive care for all age groups can help improve the oral health status of insured individuals and reduce the financial burden for both insurers and policyholders. Additionally, our study demonstrated that data mining approaches, such as regression models, are useful for identifying patterns of service utilization, predicting customer demand and choices, and planning future strategies using extensive insurance datasets. Further research is needed to evaluate the utilization of services in conjunction with the oral health conditions of insured individuals. The findings of such research could shed light on the impact of owning complementary dental insurance on the oral health status of individuals and communities.

## Data Availability

The datasets used and/or analyzed during the current study are available through the corresponding author on reasonable request.

## Abbreviations

ICD: International Classification of Diseases
CIO: Central Insurance Organization
CRISP-DM: Cross-Industry Standard Process for Data Mining
